# Associations between multimorbidity and additional burden for working-age adults with specific forms of musculoskeletal conditions: a cross-sectional study

**DOI:** 10.1186/s12891-017-1496-2

**Published:** 2017-04-04

**Authors:** Dianne B. Lowe, Michael J. Taylor, Sophie J. Hill

**Affiliations:** 1 Centre for Health Communication and Participation, School of Psychology and Public Health, College of Science, Health and Engineering, La Trobe University, Melbourne, Australia; 2 School of Allied Health, Australian Catholic University, Fitzroy, Australia; 3 Cochrane Consumers and Communication Review Group, School of Psychology and Public Health, College of Science, Health and Engineering, La Trobe University, Melbourne, Australia

**Keywords:** Multimorbidity, Musculoskeletal conditions, Quality of life, Healthcare utilisation

## Abstract

**Background:**

Multiple health conditions are increasingly a problem for adults with musculoskeletal conditions. However, multimorbidity research has focused primarily on the elderly and those with a limited subset of musculoskeletal disorders. We sought to determine whether associations between multimorbidity and additional burden differ with specific forms of musculoskeletal conditions among working-age adults.

**Methods:**

Data were sourced from a nationally representative Australian survey. Specific musculoskeletal conditions examined were osteoarthritis; inflammatory arthritis; other forms of arthritis or arthropathies; musculoskeletal conditions not elsewhere specified; gout; back pain; soft tissue disorders; or osteoporosis. Multimorbidity was defined as the additional presence of one or more of the Australian National Health Priority Area conditions. Burden was assessed by self-reported measures of: (i) self-rated health (ii) musculoskeletal-related healthcare and medicines utilisation and, (iii) general healthcare utilisation. Associations between multimorbidity and additional health or healthcare utilisation burden among working-age adults (aged 18 – 64 years of age) with specific musculoskeletal conditions were estimated using logistic regression, adjusting for confounders. Interaction terms were fitted to identify whether there were specific musculoskeletal conditions where multimorbidity was more strongly associated with poorer health or greater healthcare utilisation than in the remaining musculoskeletal group.

**Results:**

Among working-age adults, for each of the specified musculoskeletal conditions, multimorbidity was associated with similar, increased likelihood of additional self-rated health burden and certain types of healthcare utilisation. While there were differences in the relationships between multimorbidity and burden for each of the specific musculoskeletal conditions, no one specific musculoskeletal condition appeared to be consistently associated with greater additional health burden in the presence of multimorbidity across the majority of self-rated health burden and healthcare use measures.

**Conclusions:**

For working-age people with any musculoskeletal conditions examined here, multimorbidity increases self-reported health and healthcare utilisation burden. As no one musculoskeletal condition appears consistently worse off in the presence of multimorbidity, there is a need to better understand and identify strategies that acknowledge and address the additional burden of concomitant conditions for working-age adults with a range of musculoskeletal conditions.

## Background

Musculoskeletal conditions are a grouping of clinically distinct but characteristically painful conditions affecting joints, bones and muscles. Collectively, musculoskeletal conditions have one of the highest population prevalence of any chronic conditions [1]. Although musculoskeletal conditions are relatively heterogeneous and differ in aetiology and prognosis, these conditions are generally progressive or fluctuating in nature, and often involve complex treatments or procedures [2]. As a result, musculoskeletal conditions place a heavy health burden on individuals, health systems and society [1, 3–11]. Globally musculoskeletal conditions are the second most common cause of healthy years lost to morbidity [4, 5] with adverse impacts on quality of life [12].

Musculoskeletal conditions affect all age groups, but are more common with increasing age. Nonetheless, nearly one in five working-age adults are diagnosed with a musculoskeletal condition [13–15]. For working-age adults, musculoskeletal conditions pose additional employment, reproductive health, social and financial challenges [3, 16–19].

People with musculoskeletal conditions are often living with additional chronic conditions alongside [13, 14, 17, 20–30]. Chronic co-occurring conditions are termed *multimorbidity* [20, 23], or in the context of a primary condition, *comorbidity* [14, 20, 31]. Like with musculoskeletal conditions, multimorbidity also increases health expenditure [32], usage of healthcare services, polypharmacy, premature mortality, and negatively impacts physical functioning and quality of life [28, 33–37]. These factors suggest the need for clinical and policy efforts to adapt to challenges faced by working-age people with musculoskeletal conditions by considering the possible impacts of other conditions [2, 22, 24, 26–28, 32]. With ageing populations in many developed countries, delayed retirement has been advocated as a possible strategy for sustaining economic growth [38]. Both longevity and delayed retirement could have implications on workforce participation for the increasing numbers of adults experiencing magnified physical disability and reduced quality of life from accumulating conditions alongside musculoskeletal conditions [30, 38, 39].

We previously examined the prevalence of and associations between multimorbidity and self-rated health or healthcare burden measures among working-age adults with any musculoskeletal condition when using three different ways of defining multimorbidity [40, 41]. Regardless of the definition used, multimorbidity was highly associated with the majority of the health burden measures within the combined musculoskeletal population [41]. These findings highlight the level of additional self-rated health and healthcare utilisation burden associated with multimorbidity within the broad working-age population with any musculoskeletal condition.

There are a number of gaps in the existing evidence base for the relationships between multimorbidity and additional burden in those with musculoskeletal conditions. Both multimorbidity and musculoskeletal condition research usually examines older populations (i.e., aged 65 years and over), and these populations are predominately sampled within clinical settings, rather than being population representative. Additionally, when multimorbidity research includes musculoskeletal conditions it sometimes focuses exclusively on a singular musculoskeletal condition (e.g. osteoarthritis [42]) or a limited subset (e.g. arthritis conditions [29], and rheumatic conditions [30]), rather than including the breadth of musculoskeletal conditions. Particularly overlooked musculoskeletal conditions in the context of multimorbidity research include chronic back pain, gout, and osteoporosis. Furthermore, when determining the self-rated health burden of multimorbidity, research tends to combine musculoskeletal conditions into broad umbrella categories [13–15, 21, 23, 43], and it is unclear if there are some people with specific forms of musculoskeletal conditions who are more impacted by multimorbidity than others. For these reasons, there is a lack of understanding of whether health burden associated with multimorbidity varies for specific musculoskeletal conditions.

Therefore, we sought to determine whether working-age adults with specific forms of musculoskeletal conditions are worse off in the presence of multimorbidity compared to the rest of the musculoskeletal sample. Identifying the musculoskeletal sub-population(s) most affected by the additional burden of multimorbidity could aid priority setting, and could help determine the musculoskeletal populations in which to develop, evaluate and apply targeted interventions.

## Methods

Australia’s National Health Survey 2007–2008 (NHS 07-08) data used in this analysis were provided by the Australian Bureau of Statistics through their Microdata program [44]. The NHS 07-08 survey is completed by an adult and, where applicable, child, from each household sampled. Households are sampled by the Australian Bureau of Statistics from all Australian States and Territories and across all age groups, ensuring it is nationally representative. The questions asked, response rate, survey design and sampling strategy are reported elsewhere [44]. None of the authors were involved in the development of the questionnaire or data collection. Permission was obtained from the ABS to conduct this secondary cross-sectional analysis. La Trobe University Human Ethics Committee granted an exemption for this study as there was negligible risk involved in an analysis of previously collected, de-identified data.

For this study, the sample is NHS 07-08 respondents aged between 18 and 64 years with chronic musculoskeletal conditions. ‘Musculoskeletal conditions’, as categorised by the Australian Bureau of Statistics, included: osteoarthritis; inflammatory arthritis; other forms of arthritis or arthropathies; musculoskeletal conditions not elsewhere specified; gout; back pain; soft tissue disorders; and osteoporosis. The exposure is multimorbidity operationalised as presence of one or more of the following Australian National Health Priority Area condition categories (in addition to a musculoskeletal condition): diabetes; cancer; cardiovascular disease; asthma; chronic obstructive pulmonary disease; and mental health disorders [45]. Both the musculoskeletal conditions and the condition categories included in the multimorbidity variable were “chronic”, i.e. current and present for at least 6 months. Conditions were identified through self-reports of doctor diagnoses in response to open ended questions in combination with a series of prompts for some specific conditions, designed to provide respondents with the opportunity to report detailed information on all chronic conditions they experience. All chronic conditions reported were then coded by the Australian Bureau of Statistics to a categorisation system based on the Tenth Revision of the International Classification of Diseases and Health Related Problems (ICD-10-AM). Further details on the specific diagnoses included in each condition category that contributed to multimorbidity are published elsewhere [40, 41].

For each specific musculoskeletal condition, we examined the associations between the exposure (multimorbidity) and a range of burden measures. See Table 1 for the description of these burden measures.

**Table 1:** Description of burden measures

Burden Measure	Description	Timeframe
*Self-rated health*		
Fair to poor health status	A response of either “fair” or “poor” [59] to the question “In general would you say that [your/(proxy name)] health is excellent, very good, good, fair or poor” [59].	In the four weeks prior to interview
High distress	A Kessler Psychological Distress Scale-10 (K10) score indicating high (>22 points) to very high severity of distress (30-50 points) [60].	In the four weeks prior to interview
Pain interference with activities	A response of “moderately” to “extremely” to the extent bodily pain interfered with normal work. Response options: not at all; a little bit; moderately; quite a bit; extremely and did not experience bodily pain.	In the four weeks prior to interview
Moderate to severe pain rating	A response of “moderate”, “severe” or “very severe” to the extent of bodily pain felt. Response options: none; very mild; mild; moderate; severe; or very severe.	In the four weeks prior to interview
*Musculoskeletal-related* ^a^ *healthcare and medicines utilisation*		
Visit general practitioner (GP) for arthritis or osteoporosis	Self-reports of arthritis or osteoporosis-related GP consultations.	In the two weeks prior to the interview
Visit arthritis or osteoporosis specialist(s)	Self-reports of arthritis or osteoporosis-related specialist consultations.	In the two weeks prior to the interview
Pain medicines use	Self-reports of pain medicines use including analgesics.	In the two weeks prior to the interview
Musculoskeletal (MSK) medicines use	Self-reports of up to three MSK-related, main pharmaceutical medicines used. This included vitamins and mineral supplements such as vitamin D, calcium, glucosamine and various marine-based products, natural or herbal treatments.	In the two weeks prior to the interview
*General healthcare utilisation*		
Visit other specialist(s)	Self-reports of consulting a specialist for condition other than arthritis or osteoporosis.	In the 12 months prior to the interview
Visit physiotherapist(s)	Self-reports of consulting a physiotherapist.	In the 12 months prior to the interview
Did not visit health professional (HP).	Self-reports of not consulting a health professional.	In the 12 months prior to the interview
Visit chemist(s) for advice only	Self-reports of consulting a chemist for advice only (i.e., not to fill scripts).	In the 12 months prior to the interview
Visit chiropractor(s)	Self-reports of consulting a chiropractor.	In the 12 months prior to the interview

^a^All musculoskeletal-related healthcare, pain and medicines use data were collected only for NHS respondents with current arthritis or osteoporosis (osteoporosis, osteopenia or gout, rheumatism or arthritis, osteoarthritis, rheumatoid arthritis, or other type (specified))

### Analyses

To determine whether working-age adults with some specific forms of musculoskeletal conditions are worse off in the presence of multimorbidity, compared to the rest of the musculoskeletal sample, we conducted three types of analyses.

1. Prevalence of conditions comprising multimorbidity in working-age Australians with specific musculoskeletal conditions was estimated. To account for the survey design, the replicant weights generated by the Australian Bureau of Statistics were applied using the jack-knife method [46], allowing the prevalence estimates to be representative of the Australian population.
2. Associations between multimorbidity and additional health or healthcare utilisation burden among working-age adults with specific musculoskeletal conditions were estimated. Although we considered an ordinal logistic regression model for the variables “fair to poor health status” and “pain limiting work”, because the proportional odds assumption did not hold, it was not possible to treat these variables as ordered categories. It was also not possible to analyse the Kessler Psychological Distress Scale score as a continuous outcome as the data were not normally distributed, and it was not possible to transform the data to approximate normality. Therefore, the associations between multimorbidity and each burden measure among people with the specific nominated musculoskeletal condition were estimated using logistic regression. In each case, the dependent variable was the burden measure (see Table 1), and multimorbidity was the independent variable. These associations were estimated in the total working-age sample with musculoskeletal conditions, and then stratified by each specific musculoskeletal condition grouping (regardless of the presence of other musculoskeletal conditions).
3. Interaction terms were fitted to identify if the associations between multimorbidity and the various burden measures differed between the specific musculoskeletal conditions. To do this, the analysis was restricted to the working-age sample with any form of musculoskeletal condition, and the interaction term was fitted between multimorbidity and the presence of the specific musculoskeletal condition grouping. Significant (*p* < 0.05) interaction terms would indicate a greater (or lesser) burden of multimorbidity for a specific musculoskeletal condition compared to the rest of the musculoskeletal sample.

Logistic regression and interaction analyses were adjusted for age, gender, household income quintiles as a marker of socioeconomic status (SES), education, smoking status (current, past, never), and moderate to high risky alcohol consumption as potential confounders. All statistical analyses were performed using Stata (release 10.1, College Station, TX).

## Results

Of the 20,788 people interviewed from the 15,792 households sampled (a response rate of 91%), 12,604 people were working-aged (18 to 64 years). Of the working-age adults, 50.1% were female and 37.0% were aged between 18 and 34 years, 34.6% were aged between 35 and 59 years, and 28.3% were 60 to 64 years old. Chronic musculoskeletal conditions were reported by 36.1% of the working-age adults. Back pain was the most common (19.8%), followed by osteoarthritis (7.9%); other arthritis or arthropathies (8.1%); gout (4.7%); soft tissue disorders (3.2%); inflammatory arthritis (2.5%); osteoporosis (2.3%) or other musculoskeletal conditions not elsewhere specified (0.9%). The prevalence of multimorbidity among working-age adults was 15.3% (Table 2). Among the working-age adults, the prevalence of the chronic conditions included in the multimorbidity definition were as follows: mental health disorders 13.4%; asthma 9.7%; cardiovascular diseases (CVD) 5.2%; diabetes 3.4%; chronic obstructive pulmonary disease (COPD) 2.1%; and cancer 1.4% (Table 2).

**Table 2:** Prevalence^a^ of conditions comprising multimorbidity in working-age Australians with specific musculoskeletal conditions

Sample (% of working-age, *n* = 12,604)	Cancer (1.6%) % (95%CI)	Diabetes (3.4%) % (95%CI)	CVD (5.5%) % (95%CI)	Asthma (10.6%) % (95%CI)	COPD (2.5%) % (95%CI)	MH disorder (14.5%) % (95%CI)	Any condition (17.0%) % (95%CI)	Median # of these conditions^b^ (10th-90th percentile)
Osteoarthritis (7.9%)	4.4 (2.6,6.2)	7.9 (5.1,10.7)	14.7 (11.0,18.3)	14.5 (11.7,17.4)	6.0 (4.3,7.6)	21.5 (17.7,25.4)	46.1 (41.2,51.0)	1 (1,3)
Inflammatory arthritis (2.5%)	3.7 (1.0,6.3)	11.3 (5.6,17.0)	15.6 (10.3,21.0)	19.3 (13.8,24.8)	7.0 (3.2,10.9)	23.5 (17.0,29.9)	53.1 (45.0,61.1)	1 (2,3)
Other arthritis (8.1%)	3.0 (1.5,4.5)	7.9 (5.4,10.4)	11.5 (8.4,14.7)	14.4 (11.7,17.1)	4.4 (2.8,6.0)	19.5 (16.6,22.4)	41.9 (38.0,45.7)	1 (1,3)
Other MSK (0.9%)	3.6 (0.0,8.0)	2.5 (0.0,5.7)	14.7 (7.4,21.9)	7.0 (1.8,12.1)	5.1 (0.4,9.9)	26.2 (13.1,39.3)	41.4 (26.9,55.9)	1 (1,3)
Gout (4.7%)	2.9 (1.0,4.9)	9.3 (6.1,12.5)	10.0 (7.3,12.7)	8.8 (5.7,11.9)	2.7 (1.3,4.0)	16.6 (12.3,20.9)	37.8 (32.7,43.0)	1 (1,3)
Back pain (19.8%)	1.8 (1.2,2.5)	5.2 (3.8,6.5)	8.4 (6.8,10.0)	12.7 (11.1,14.3)	4.6 (3.7,5.5)	22.6 (20.5,24.8)	39.8 (37.6,42.0)	1 (1,3)
Soft tissue disorder (3.2%)	2.9 (1.1,4.6)	8.1 (3.8,12.3)	14.4 (9.0,19.8)	16.7 (12.6,20.9)	4.3 (1.9,6.8)	22.7 (17.5,28.0)	46.9 (40.2,53.6)	1 (1,3)
Osteoporosis (2.3%)	5.5 (1.8,9.1)	5.5 (2.2,8.7)	15.7 (8.4,23.0)	15.4 (10.1,20.7)	7.7 (4.0,11.4)	23.4 (18.3,28.5)	50.4 (42.0,58.8)	1 (2,3)
Any MSK (36.1%)	2.3 (1.8,2.9)	5.8 (4.8,6.8)	9.3 (8.0,10.6)	12.1 (10.9,13.4)	4.0 (3.3,4.6)	19.2 (17.6,20.7)	38.3 (36.5,40.1)	1 (1,3)
Working-age all (100%)	1.4 (0.1,1.7)	3.4 (2.9,3.9)	5.2 (4.5,5.9)	9.7 (9.1,10.3)	2.1 (1.8,2.3)	13.4 (12.8,14.1)	15.3 (14.3,16.2)	0 (0,2)

legend: ^a^Prevalence estimates take into account survey design by using the jack-knife method to apply the replicant weights generated by the Australian Bureau of Statistics. MSK: musculoskeletal conditions. CVD: cardiovascular disease. COPD: chronic obstructive pulmonary disease﻿. MH disorder: mental health disorder. Any condition: at least one of cancer, diabetes, asthma, COPD or MH disorder. ^b^Including MSK

Depending on the specific musculoskeletal condition grouping, the prevalence of multimorbidity varied from 37.8% (among those with gout) to 53.1% (among those with inflammatory arthritis) (Table 2). The three most common comorbidities alongside any musculoskeletal condition were typically a mental health disorder, followed by asthma or CVD. There were minimal differences in the frequency pattern of co-occurring conditions for working-age adults with specific musculoskeletal conditions (Table 2), excepting for those with gout, where diabetes was more common than asthma. Furthermore, although cancer was typically the least common co-occurring condition alongside the specific musculoskeletal conditions, for those with gout, COPD was least common, and for those with for those with “other musculoskeletal conditions” diabetes was the least common co-morbidity. Among working-age adults with specific musculoskeletal conditions, 35.7 to 69.1% also reported the presence of an additional musculoskeletal condition (Table 3) (lowest among those with back pain and highest among those with osteoporosis).

**Table 3:** Prevalence of additional musculoskeletal conditions in working-age Australians^a^ with specific musculoskeletal conditions

Sample (n)	Osteo-arthritis % (95%CI)	Inflammatory arthritis % (95%CI)	Other arthritis % (95%CI)	Other MSK % (95%CI)	Gout % (95%CI)	Back pain % (95%CI)	Soft tissue conditions % (95%CI)	Osteo-porosis % (95%CI)	Additional MSK % (95%CI)	Median # of MSK conditions (10th-90th percentile)
Osteoarthritis (993)	100	3.6 (1.9, 5.4)	6.3 (3.9, 8.6)	2.1 (0.8, 3.3)	7.5 (5.6, 9.4)	33.3 (28. 8, 37.9)	8.4 (5.7, 11.1)	10.7 (8.0, 13.4)	52.1 (48.2, 56.0)	1 (2,3)
Inflammatory arthritis (317)	11.8 (6.5, 17.0)	100	4.6 (1.6, 7.7)	3.3 (1.1, 5.5)	10.2 (6.0, 14.5)	37.3 (29.4, 45.2)	24.7 (17.7, 31.8)	6.2 (3.4, 9.1)	63.2 (54.5, 72.0)	1 (2,3)
Other arthritis (1022)	5.7 (3.7, 7.6)	1.3 (0.4, 2.1)	100	1.6 (0.3, 2.8)	6.3 (4.5, 8.2)	33.3 (29.6, 36.9)	6.1 (4.3, 8.0)	4.9 (3.3, 6.6)	46.3 (42.5, 50.1)	1 (1,3)
Other MSK (117)	18.0 (7.8, 28.3)	8.7 (2.9, 14.5)	14.9 (4.1, 25.6)	100	7.2 (1.9, 12.4)	31.5 (20.4, 42.6)	7.6 (0.5, 14.6)	10.0 (3.2, 16.8)	63.5 (51.7, 75.2)	1 (2,3)
Gout (589)	11.8 (8.6, 14.9)	5.0 (2.7, 7.2)	11.0 (7.8, 14.2)	1.3 (0.2, 2.4)	100	27.4 (22.6, 32.3)	8.1 (5.3, 10.8)	2.8 (1.3, 4.4)	46.3 (40.9, 51.8)	1 (1,3)
Back pain (2493)	12.1 (10.2, 14.0)	4.2 (3.0, 5.3)	13.3 (11.5, 15.1)	1.3 (0.8, 1.9)	6.3 (5.2, 7.5)	100	5.8 (4.5, 7.1)	3.7 (2.7, 4.7)	35.7 (33.0, 38.5)	1 (1,2)
Soft tissue disorder (404)	18.4 (12.6, 24.1)	16.7 (11.7, 21.7)	14.8 (10.7, 19.0)	1.9 (0.1, 3.7)	11.2 (7.6, 14.9)	34.9 (29.0, 40.8)	100	8.6 (4.6, 12.6)	62.4 (56.3, 68.6)	1 (2,4)
Osteoporosis (292)	33.9 (26.6, 41.2)	6.1 (3.1, 9.1)	17.2 (11.2, 23.2)	3.7 (1.2, 6.2)	5.7 (2.7, 8.8)	32.6 (24.7, 40.4)	12.5 (6.9, 18.1)	100	69.1 (62.0, 76.3)	1 (2,4)
Any MSK (4555)	20.1 (18.8, 21.5)	6.2 (5.3, 7.1)	22.2 (20.3, 24.1)	2.3 (1.7, 3.0)	12.8 (11.7, 13.9)	55.5 (53.3, 57.6)	9.2 (7.9, 10.5)	6.3 (5.5, 7.2)	27.4 (25.7, 29.0)	1 (1,2)

legend: ^a^Prevalence estimates take into account survey design by using the jack-knife method to apply the replicant weights generated by the Australian Bureau of Statistics. MSK: musculoskeletal conditions

### Musculoskeletal condition, multimorbidity and self-rated health burden

Among working-age adults, multimorbidity was consistently associated with greater additional self-rated health burden, with minimal differences across these specific musculoskeletal conditions (Table 4). There were insufficient numbers in the “other musculoskeletal” condition group to calculate valid estimates; therefore results are not presented in Tables 4, 5 and 6. Across the specific musculoskeletal conditions, while there were some differences in association between multimorbidity and self-rated health burden measures, these differences were mostly inconsistent and estimates were imprecise, due to small sample sizes for some specific musculoskeletal conditions.

**Table 4:** Associations^a^ and interactions^b^ between multimorbidity and self-reported health measures among working-age adults with specific musculoskeletal conditions

Sample (*n* = 4555)	Fair to poor health status (*n* = 1073) OR (95%CI)	Interaction *p*-value^b^	High distress (*n* = 865) OR (95%CI)	Interaction *p*-value	Pain interference with activities (*n* = 1633) OR (95%CI)	Interaction *p*-value	Moderate to severe pain (*n* = 2086) OR (95%CI)	Interaction *p*-value
Any MSK (4555)	3.5 (2.7, 4.4)	-	6.0 (4.8, 7.3)	-	2.0 (1.6, 2.4)	-	1.9 (1.5, 2.3)	-
Osteoarthritis (993)	3.9 (2.2, 7.0)	0.77	6.1 (3.6, 10.2)	0.53	1.8 (1.2, 2.8)	0.56	2.1 (1.3, 3.3)	0.94
Inflammatory arthritis (317)	3.9 (1.4, 10.4)	0.27	5.4 (2.1, 14.0)	0.52	2.9 (1.3, 6.8)	0.43	2.3 (1.0, 5.3)	0.55
Other arthritis (1022)	3.4 (1.9, 5.8)	0.76	4.8 (2.8, 8.2)	0.16	1.9 (1.3, 3.0)	0.73	2.1 (1.4, 3.2)	0.82
Gout (589)	3.8 (1.8, 7.9)	0.41	7.2 (2.9, 17.9)	0.55	2.6 (1.4, 4.7)	0.54	1.7 (1.0, 2.9)	0.83
Back pain (2493)	3.8 (2.8, 5.2)	0.34	8.0 (6.0, 10.8)	**<0.01**	2.1 (1.6, 2.7)	0.13	2.0 (1.5, 2.7)	0.18
Soft tissue disorder (404)	4.0 (1.8, 9.0)	0.86	5.0 (2.0, 12.8)	0.36	3.3 (2.0, 5.6)	**<0.05**	2.1 (1.1, 3.7)	0.99
Osteoporosis (292)	5.1 (1.9, 14.0)	0.86	5.8 (2.1, 16.1)	0.37	3.4 (1.4, 8.3)	0.24	3.6 (1.5, 8.7)	0.17

legend: ^a^Each row header indicates the specific MSK in which the strata-specific association between multimorbidity and each burden measure has been estimated. For each row, the reference group is those with the specific MSK but without multimorbidity. Associations (odds ratios) are adjusted for age, gender, SES, education, smoking and medium to high risk alcohol consumption. All ratings of self-reported health burden relate to the 4 weeks prior to the interview. ^b^Each interaction *p*-value indicates whether the association between multimorbidity and the health measure differs between the specific MSK group and the remaining MSK population. Significant (*p* < 0.05) interactions are bolded and further details are presented textually in results section

**Table 5:** Associations^a^ and interactions^b^ between multimorbidity and musculoskeletal-related healthcare or medicines use among working-age adults with specific musculoskeletal conditions

Sample (*n* = 4555)	Visit arthritis or osteoporosis specialist (*n* = 103) OR (95%CI)	Interaction *p*-value^b^	Visit GP for arthritis or osteoporosis (*n* = 206) OR (95%CI)	Interaction *p*-value	Pain medicines use (*n* = 238) OR (95%CI)	Interaction *p*-value	MSK medicines use (*n* = 1179) OR (95%CI)	Interaction *p*-value
Any MSK (4555)	1.4 (0.7, 2.8)	-	1.4 (1.0, 2.2)	-	3.2 (2.1, 5.1)	-	1.3 (1.1, 1.7)	-
Osteoarthritis (993)	1.7 (0.5, 5.6)	0.68	2.1 (1.0, 4.1)	0.30	3.5 (1.7, 7.5)	0.91	1.3 (1.0, 1.9)	0.98
Inflammatory arthritis (317)	0.8 (0.1, 4.9)	0.59	0.7 (0.1, 6.1)	0.43	2.9 (0.7, 12.2)	0.51	0.8 (0.3, 1.9)	0.08
Other arthritis (1022)	1.3 (0.5, 3.2)	0.79	2.3 (0.9, 6.1)	0.21	2.1 (1.2, 3.8)	**<0.05**	1.0 (0.7, 1.6)	0.11
Gout (589)	NE	NE	20.8 (2.8, 157.9)	0.84	10.0 (1.4, 71.8)	0.40	2.4 (1.2, 5.1)	**<0.05**
Back pain (2493)	3.0 (0.7 13.9)	0.23	2.6 (1.0, 7.2)	0.14	3.9 (2.0, 7.8)	0.40	1.7 (1.2, 2.3)	0.06
Soft tissue disorder (404)	1.4 (0.0, 164.0)	0.93	2.6 (0.7, 10.2)	0.34	4.0 (1.0, 16.0)	0.96	2.2 (1.2, 4.0)	0.17
Osteoporosis (292)	1.0 (0.4 2.4)	0.92	1.0 (0.4, 2.1)	0.94	3.4 (0.9, 13.8)	0.68	1.6 (0.8, 3.4).	0.91

legend: ^a^Each row header indicates the specific MSK in which the strata-specific association between multimorbidity and each burden measure has been estimated. For each row, the reference group is those with the specific MSK but not multimorbidity. Associations (odds ratios) are adjusted for age, gender, SES, education, smoking and medium to high risk alcohol consumption. All musculoskeletal-related healthcare and medicines use measures relate to the 2 weeks prior to the interview. NE: not estimable due to small cell counts. ^b^Each interaction *p*-value indicates whether the association between multimorbidity and the health measure differs between the specific MSK group and the remaining MSK population. Significant (*p* < 0.05) interactions are bolded and further details are presented textually in results section. GP: general practitioner

**Table 6:** Associations^a^ and interactions^b^ between multimorbidity and general healthcare utilisation among working-age adults with specific musculoskeletal conditions

Sample (*n* = 4555)	Visit other specialist (*n* = 1513) OR (95% CI)	Interaction *p*-value^b^	Visit physiotherapist (*n* = 665) OR (95% CI)	Interaction *p*-value	Did not visit HP (*n* = 1422) OR (95% CI)	Interaction *p*-value	Visit chemist for advice only (*n* = 779) OR (95% CI)	Interaction *p*-value	Visit chiropractor (*n* = 664) OR (95% CI)	Interaction *p*-value
Any MSK (4555)	2.3 (2.0, 2.7)	-	1.1 (0.9, 1.5)	-	0.4 (0.3, 0.5)	-	2.0 (1.7, 2.5)	-	1.0 (0.7, 1.2)	-
Osteoarthritis (993)	2.4 (1.7, 3.3)	0.81	1.3 (0.7, 2.3)	0.82	0.5 (0.3, 0.9)	0.20	1.8 (1.1, 3.0)	0.61	0.7 (0.3, 1.4)	0.55
Inflammatory arthritis (317)	0.9 (0.4, 2.1)	**<0.01**	0.9 (0.3, 3.1)	0.85	0.7 (0.3, 1.3)	0.16	4.9 (2.1, 11.4)	**<0.05**	0.8 (0.2, 3.2)	0.64
Other arthritis (1022)	2.6 (1.7, 3.8)	0.68	1.0 (0.6, 1.8)	0.48	0.4 (0.2, 0.5)	0.86	1.6 (1.0, 2.6)	0.08	0.9 (0.5, 1.5)	0.79
Gout (589)	3.6 (2.1, 6.1)	0.13	1.2 (0.5, 2.9)	0.91	0.3 (0.2, 0.5)	0.31	1.9 (1.0, 3.7)	0.89	0.6 (0.4, 2.0)	0.55
Back pain (2493)	2.8 (2.1, 3.5)	0.05	1.1 (0.8, 1.4)	0.83	0.4 (0.3, 0.5)	0.80	1.8 (1.3, 2.4)	0.36	0.9 (0.6, 1.1)	0.28
Soft tissue disorder (404)	2.6 (1.4, 4.8)	0.85	1.1 (0.5, 2.4)	0.95	0.3 (0.2, 0.8)	0.55	3.5 (1.3, 9.3)	0.07	0.4 (0.2, 0.9)	0.10
Osteoporosis (292)	2.4 (0.9, 6.1)	0.90	1.3 (0.3, 5.4)	0.91	0.4 (0.1, 1.3)	0.88	2.1 (0.7 6.4)	0.95	0.7 (0.2, 2.4)	0.31

legend: ^a^ Each row header indicates the specific MSK in which the strata-specific association between multimorbidity and each burden measure has been estimated. For each row, the reference group is those with the specific MSK but not multimorbidity. Associations (odds ratios) are adjusted for age, gender, SES, education, smoking and medium to high risk alcohol consumption. All general healthcare utilisation measures relate to the 12 months prior to the interview. ^b^Each interaction *p*-value indicates whether the association between multimorbidity and the burden measure differs between the specific MSK group and the remaining MSK population. Significant (*p* < 0.05) interactions are bolded and further details are presented textually in results section

However, interaction analyses between each specific musculoskeletal condition and multimorbidity for each of the self-rated health burden measures identified two instances of significant between group differences. Firstly, the relationship between multimorbidity and the measure “high distress” was significantly greater in those with back pain compared to the remainder of the musculoskeletal group (Table 4). Specifically, of working-age adults with chronic back pain (*n* = 2493), a lower proportion (8.0% *n* = 117/1467) without multimorbidity reported high distress compared to 38.8% (*n* = 398/1025) who had multimorbidity (OR = 8.0, 95%CI: 6.0, 10.8). In those with another form of musculoskeletal condition (other than back pain, *n* = 2061) those with multimorbidity also reported greater rates of high distress (30.7%, 247/806) compared to those without multimorbidity (8.2%, *n* = 103/1255, OR = 4.1, 95%CI: 3.0, 5.5). Therefore, although multimorbidity is a risk factor for high distress in all forms of musculoskeletal condition, it is a stronger risk factor in those with back pain (interaction *p*-value = 0.001). Secondly, the observed association for multimorbidity on pain interfering with work was significantly greater for those with soft-tissue disorders than the rest of the musculoskeletal group. Specifically, of working-age adults with soft-tissue disorders (*n* = 404), 37.2% (74/199) without multimorbidity reported pain interfering with work, compared to 62.4% (128/205) who had multimorbidity (OR = 3.3 95%CI: 2.0, 5.6). In those with another form of musculoskeletal condition (other than soft-tissue disorder, *n* = 4151), those with multimorbidity also reported higher rates of pain interfering with work (45.9%, 747/1627) compared to those without multimorbidity (27.1%, 684/2524, OR = 1.8 95%CI: 1.4, 2.3), but this association was weaker than that seen in those with a soft tissue disorders (interaction *p*-value = 0.03).

### Musculoskeletal condition, multimorbidity and musculoskeletal related healthcare utilisation and medicines use

After adjusting for SES, education, smoking and medium to high risk alcohol consumption (Table 5) among working-age adults with specific musculoskeletal conditions there were minimal differences in associations between the presence and absence of multimorbidity in the measures of musculoskeletal healthcare utilisation (Table 5).

The interaction analyses identified two exceptions. The association between multimorbidity and pain medicine use was weaker among those with “other arthritis” compared to those with the remaining musculoskeletal conditions (Table 5). Specifically, of working-age adults with “other arthritis” (*n* = 1022), in the absence of multimorbidity, 4.1% (*n* = 24/586) reported pain medicines use, compared to 8.0% (*n* = 35/436) when multimorbidity was present. In those with a musculoskeletal condition other than “other arthritis” (*n* = 3533), those with multimorbidity also reported higher rates of pain medicines use (8.7%, *n* = 122/1396) compared to those without multimorbidity (2.7%, *n* = 57/2137, but this association (OR = 4.0 95%CI: 2.4, 6.9) was stronger (interaction *p*-value = 0.014) than that seen in those with “other arthritis” (OR = 2.1, 95%CI: 1.2, 3.8).

The association between multimorbidity and musculoskeletal medicines use was stronger in respondents with gout than those with the other musculoskeletal conditions (Table 5). Specifically, of working-age adults with gout (*n* = 589), 15.9% (*n* = 55/345) without multimorbidity reported musculoskeletal medicines use, compared to 29.1% (*n* = 71/244) who had multimorbidity (OR = 2.4 95%CI: 1.2, 5.1). In those with another form of musculoskeletal condition (other than gout, *n* = 3966), those with multimorbidity reported higher rates of musculoskeletal medicines use (30.3%, *n* = 481/1588) compared to those without multimorbidity (24.1%, *n* = 572/2378, OR = 1.3, 95%CI: 1.0, 1.6), but this association was weaker than that seen in those with gout (interaction *p*-value = 0.040). It should be noted that this apparent stronger association in respondents with gout is due to musculoskeletal medicines use being less common (15.9%) in respondents with gout, than for the remainder of musculoskeletal conditions (24.1%) in the absence of multimorbidity. Yet, in the presence of multimorbidity there was little difference in the prevalence of musculoskeletal medicines use by respondents with gout (29.1%) and all other musculoskeletal conditions (30.3%). This indicates that, in the absence of multimorbidity, respondents with gout are less likely to use musculoskeletal medicines than those with another form of musculoskeletal condition.

### Musculoskeletal condition, multimorbidity and general healthcare utilisation

Among the combined musculoskeletal working-age population, multimorbidity was associated with consulting a specialist for conditions other than arthritis/osteoporosis; and chemist(s) for advice; but not visiting physiotherapist(s) or chiropractor(s); and was protective against not consulting a health professional during the past year (Table 6). Furthermore, there were minimal differences in the associations between multimorbidity and these general healthcare utilisation burden measures across working-age adults with the specific musculoskeletal conditions (Table 6).

Interaction analyses identified there was a greater association between multimorbidity and consulting a chemist for advice only (interaction *p*-value = 0.015) among those with inflammatory arthritis compared to those with the remaining musculoskeletal conditions (Table 6). Specifically, of working-age adults with inflammatory arthritis (*n* = 317), 14.3% without multimorbidity (*n* = 21/147) reported consulting a chemist for advice only, compared to 30.2% (*n* = 52/170) who had multimorbidity (OR = 4.9, 95%CI: 2.1, 11.4). In those with another form of musculoskeletal condition (other than inflammatory arthritis, *n* = 4238), those with multimorbidity reported higher rates of consulting a chemist for advice only (21.6%, *n* = 359/1662) than those without multimorbidity (13.5%, *n* = 347/2576, OR = 1.8, 95%CI: 1.5, 2.3), but this association was weaker than that seen in those with inflammatory arthritis (interaction *p*-value = 0.015). Additionally, multimorbidity was associated with an increased likelihood of consulting a specialist for a non-arthritis condition across the majority of the specific musculoskeletal conditions, but not in those with inflammatory arthritis. Specifically, of working-age adults with inflammatory arthritis (*n* = 317), 46.9% (*n* = 69/147) without multimorbidity reported consulting a specialist for a non-arthritis condition, compared to 56.5% (*n* = 96/170) who had multimorbidity (OR = 0.9, 95%CI: 0.4, 2.1). In contrast, in those with another form of musculoskeletal condition (other than inflammatory arthritis, *n* = 4238), those with multimorbidity reported higher rates of consulting a specialist for a non-arthritis condition (43.9%, *n* = 730/1662) than those without multimorbidity (24.0%, *n* = 618/2576, OR = 2.5, 95%CI: 2.1, 3.0), and this association was stronger than that seen in those with inflammatory arthritis (interaction *p*-value = 0.004).

## Discussion

In this population representative sample of community dwelling adults aged 18 to 64 years, for each of the specified musculoskeletal conditions there were minimal differences in associations between multimorbidity and self-rated measures of health burden or certain types of healthcare utilisation. Specifically, associations between multimorbidity and additional self-rated health, distress and pain burden measures were higher and relatively consistent across each of these specific musculoskeletal conditions. Also, for the majority of these specified musculoskeletal conditions, multimorbidity was strongly associated with the health utilisation measures of visiting ﻿general practitioner (GP) for arthritis or osteoporosis and visiting specialists for conditions other than arthritis or osteoporosis, pain and musculoskeletal medicine use, and consulting chemist for advice, but not visiting specialists for arthritis or osteoporosis, visiting physiotherapist(s) or chiropractor(s).

Despite these overall findings, interaction analyses did identify limited instances where associations between multimorbidity and some burden measures were more pronounced for some specific musculoskeletal conditions. In particular, there was a much stronger association between multimorbidity and consulting a chemist (for advice only) among those with inflammatory arthritis compared to those with the remaining musculoskeletal conditions. This may be due to inflammatory conditions, such as rheumatoid arthritis, requiring complex medication management that, in the presence of additional conditions and their respective medications, raises the possibility of drug interactions and increases the need for advice seeking. Furthermore, the relationship between multimorbidity and the measure “high distress” was significantly greater in those with back pain compared to the remainder of the musculoskeletal population. This was not simply due to pain or mental health issues being more common, as multimorbidity was not more strongly related to ratings of moderate to severe pain in those with back pain when compared to those with another form of musculoskeletal condition. Furthermore, mental health conditions were not particularly more common in those with back pain, when compared to other forms of musculoskeletal conditions. These observations may indicate that a more holistic, person centred, treatment approach for additional conditions alongside back pain may be needed. Similarly, the observed association for multimorbidity on “pain interfering with work” was significantly stronger for those with soft-tissue disorders than the rest of the musculoskeletal population, suggesting that additional support strategies for improving return to work could be indicated for individuals with soft tissue conditions alongside other conditions.

Conversely, for some specific musculoskeletal conditions, the associations between multimorbidity and some burden measures were significantly weaker. For example, associations between multimorbidity and pain medicines use were weaker for those with “other arthritis” than the remaining musculoskeletal groups. However, both the “other arthritis” group and those with the remaining musculoskeletal conditions had equivalent proportions reporting pain medicines use in the presence of multimorbidity; whereas, in the absence of multimorbidity the prevalence of pain medicines use was much higher for those with “other arthritis” than for those with the remaining musculoskeletal conditions, thus creating a weaker association. Again, in the presence of multimorbidity the proportion reporting musculoskeletal medicines use among those with gout and the remaining musculoskeletal groups were equivalent, but in the absence of multimorbidity, musculoskeletal medicines use was lower in those with gout (than the remaining musculoskeletal group), suggesting those with uncomplicated gout are less likely to use these medicines. Finally, the association between multimorbidity and consulting a specialist for a non-arthritis condition (i.e. “other specialists”) in those with “inflammatory arthritis” was significantly weaker, even though the proportion of people visiting “other specialists” in the presence of multimorbidity was higher in this group than the remaining musculoskeletal population. The association was weaker because, in the absence of multimorbidity, the proportion visiting “other specialists” was much higher in those with inflammatory arthritis than the remaining musculoskeletal population. A possible interpretation of the higher rate of visiting “other specialists” in the absence of multimorbidity is that the conditions included in policy definition do not adequately capture the experience of “other” conditions for working-age adults with inflammatory arthritis.

The associations with multimorbidity observed here are similar to those in previous studies with comparable objectives despite a focus on a limited spectrum of arthritis conditions [29], and rheumatic conditions [30] or both [39]. In the study by Mavaddat et al., who sampled from GP settings [29], the association between multimorbidity (defined as arthritis plus one of cancer, stroke, heart attack, diabetes, arthritis or respiratory illnesses) and self-rated fair to poor health was OR 1.5 (95% CI: 1.3, 1.7); and arthritis in the presence of two additional conditions was associated with a greater increase likelihood of self-rated fair to poor health 3.0 (95% CI: 2.1-4.1). In Loza et al.’s study, when multimorbidity included a rheumatic condition, subjective health-related quality of life and daily functioning was worse than multimorbidity that did not include a rheumatic condition [30]. Similar to our findings, co-morbidity negatively impacted physical and mental health equally according to those with either rheumatoid arthritis or osteoarthritis [39]. Collectively, these results support the finding of additional burden caused by multimorbidity on individuals with musculoskeletal conditions. Our finding that the proportions of the working-age adults with specific additional conditions did not vary greatly across the specific musculoskeletal conditions supports previous research that similarly found that the proportions of the arthritis sample with particular comorbidities did not vary substantially with the type of arthritis (osteoarthritis, rheumatoid arthritis, rheumatism, other arthritis) [47].

Within this study, we elected to define multimorbidity as the presence of one or more of the Australian National Health Priority Area conditions [45] in addition to a musculoskeletal condition (listed above). We have termed this the “policy definition” of multimorbidity [40, 41]. As there isn’t a gold standard for operationalising multimorbidity based on condition counts, we have previously examined three potential definitions. While these definitions resulted in highly variable estimates of prevalence of multimorbidity [40], all definitions examined were similar in terms of associations with burden [41]. However, the policy definition was more strongly associated with distress [41]. We elected to use the policy definition of multimorbidity within this study, as it produced an intermediate estimate of prevalence, and generated similar strengths of associations with subjective health outcomes among the musculoskeletal population as the other assessed definitions. If we had chosen a different definition, the specific association estimates would have been different, but it is unlikely that this would have materially altered the study findings due to the inherent nature of dichotomous cut-points flattening associations [41]. The associations reported for the overall musculoskeletal group are slightly different than in a previous paper [41], as we adjusted here for additional factors beyond age, gender as potential confounders (household income quintiles as a marker of socioeconomic status (SES), education, smoking status (current, past, never), and moderate to high risky alcohol consumption). Adjusting for the additional factors generally resulted in reducing the strength of associations (by no more than 15%), but did not change the direction or interpretation in any instance.

This study has a number of strengths and limitations. Although we observe interactions, given a large number of exploratory analyses were performed, it is possible that some of the observed interactions are spurious. Given the multiple testing and the age of the dataset, these analyses should be replicated in future research. The strengths of this study are that it is nationally representative, with a large sample size, and a comprehensive range of subjective measures of health status, distress, pain, healthcare utilisation, and medicines use. This allowed a comprehensive approach to examining the additional health burden of multimorbidity for working-age adults with a range of musculoskeletal conditions. While the total sample size was quite large, the “other musculoskeletal” group was small in this sample, resulting in imprecise estimates of associations for the associations between multimorbidity and the health status and utilisation measures. For this reason, we elected not to present these results. A larger population sample, or a selected clinical sample, may be required to address this issue in this sub-population. Both the exposures (report of doctor diagnosis of conditions) and outcomes (health and healthcare utilisation measures) were based on participant recall. Reported conditions were therefore salient to the respondents, but may potentially be subject to recall error. Recall error may overestimate associations because identifying symptom-based conditions may also mean those with poorer health may recall more symptoms. However, there is no clear reason to believe that this recall bias should be different between people with specific musculoskeletal conditions or for those with or without multimorbidity, which is the focus of this study. Additionally, a limitation of using population-based survey data is that self-report of arthritis may not be discriminating enough to allow for exploration of within sub-group analyses [47]. The cross-sectional design of this study does not allow for examination of temporality of associations. However, given poor quality of life does increase risk of depression and chronic pain, longitudinal studies are required to examine the temporality of poor health status and multimorbidity or musculoskeletal conditions.

This study extends previous research examining associations between multimorbidity and health status and utilisation measures to determine whether there is a particular musculoskeletal sub-population for whom the additional burden of multimorbidity is most pronounced. We found that the additional health burden of multimorbidity is an issue for all of these specific musculoskeletal conditions and this highlights the need for proactive healthcare management, such as contemporary models of care, whereby the principles of chronic care are integrated within an interdisciplinary team that supports and involves the patient in their care planning [48]. A range of strategies to deal with multimorbidity in health services have been evaluated [49, 50]. However, intervention trials in these reviews are focused on multimorbidity (or common combinations of comorbidities) that do not include a musculoskeletal condition and populations rarely include those aged less than 65 years [49, 50]. Nonetheless, some strategies might also be relevant to all adults with multimorbidity as a way of improving care continuity or optimising care or treatments by tailoring them to meet a person's needs [49]. In these reviews, a limited number of trials are of some relevance to multimorbidity/comorbidity that includes a musculoskeletal condition with only a subset of the population under the age of 65 years, these interventions show some limited, small or mixed benefits across a range of health outcomes [51–55]. It is therefore perhaps unsurprising that adults and younger people with musculoskeletal conditions [56, 57] identify a number of unmet healthcare service and information needs. These unmet needs can help inform improvements to existing models of care and furthermore emphasise the need to involve consumers in implementation of models of care. Promising strategies for involving consumers when designing and delivering models of care, include consumer involvement in: developing guidelines, identifying barriers to implementation and tailoring implementation strategies [58]. By focusing on working-age adults, we extend previous research, which has focused primarily on older people. Our findings emphasise that multimorbidity is associated with additional impact among working-age people. We were interested in the subjective burden of multimorbidity compared to the subjective burden of musculoskeletal conditions. So, while the multimorbid musculoskeletal population may also be considered comorbid we use the term multimorbidity as we are not technically applying an index condition, rather, the sample is the musculoskeletal population, and the exposure is the presence of multimorbidity.

## Conclusions

Among working-age adults with any musculoskeletal conditions examined here, multimorbidity was associated with similar increases in all self-reported health and some healthcare utilisation burden measures. The implication of these findings is that there is little rationale, based on health status or health utilisation burden, for directing interventions to improve outcomes of individuals with multimorbidity to only people with a specific musculoskeletal condition. The additional burden of multimorbidity among the working-age population with a range of musculoskeletal conditions highlights the need to better understand and identify strategies that acknowledge and address the issues of concomitant conditions. However, an approach targeted to those with only specific musculoskeletal conditions at the exclusion of all others could arguably worsen burden associated with multimorbidity or be otherwise inequitable.

## Abbreviations

CVD: Cardiovascular disease
COPD: Chronic obstructive pulmonary disease
GP: General practitioner
ICD-10-AM: International Statistical Classification of Diseases and Related Health Problems, Tenth Revision, Australian Modification
MH disorder: Mental health disorder
MSK: Musculoskeletal condition
NHS: National health survey
SES: Socioeconomic status
